# The CECARI Study: Everolimus (Certican®) Initiation and Calcineurin Inhibitor Withdrawal in Maintenance Heart Transplant Recipients with Renal Insufficiency: A Multicenter, Randomized Trial

**DOI:** 10.1155/2017/6347138

**Published:** 2017-02-20

**Authors:** Jan Van Keer, David Derthoo, Olivier Van Caenegem, Michel De Pauw, Eric Nellessen, Nathalie Duerinckx, Walter Droogne, Gábor Vörös, Bart Meyns, Ann Belmans, Stefan Janssens, Johan Van Cleemput, Johan Vanhaecke

**Affiliations:** ^1^Department of Cardiology, University Hospitals Leuven, 3000 Leuven, Belgium; ^2^Department of Cardiology, Cliniques Universitaires Saint-Luc, 1200 Bruxelles, Belgium; ^3^Department of Cardiology, University Hospitals Ghent, 9000 Ghent, Belgium; ^4^Department of Cardiology, Centre Hospitalier Universitaire de Liège-Sart Tilman, 4000 Liège, Belgium; ^5^Department of Cardiac Surgery, University Hospitals Leuven, 3000 Leuven, Belgium; ^6^Interuniversity Institute for Biostatistics and Statistical Bioinformatics, Hasselt University & Catholic University of Leuven, 3000 Leuven, Belgium

## Abstract

In this 3-year, open-label, multicenter study, 57 maintenance heart transplant recipients (>1 year after transplant) with renal insufficiency (eGFR 30–60 mL/min/1.73 m^2^) were randomized to start everolimus with CNI withdrawal (*N* = 29) or continue their current CNI-based immunosuppression (*N* = 28). The primary endpoint, change in measured glomerular filtration rate (mGFR) from baseline to year 3, did not differ significantly between both groups (+7.0 mL/min in the everolimus group versus +1.9 mL/min in the CNI group, *p* = 0.18). In the on-treatment analysis, the difference did reach statistical significance (+9.4 mL/min in the everolimus group versus +1.9 mL/min in the CNI group, *p* = 0.047). The composite safety endpoint of all-cause mortality, major adverse cardiovascular events, or treated acute rejection was not different between groups. Nonfatal adverse events occurred in 96.6% of patients in the everolimus group and 57.1% in the CNI group (*p* < 0.001). Ten patients (34.5%) in the everolimus group discontinued the study drug during follow-up due to adverse events. The poor adherence to the everolimus therapy might have masked a potential benefit of CNI withdrawal on renal function.

## 1. Introduction

Calcineurin inhibitors (CNIs) have made an invaluable contribution to the improvement of short and mid-term survival after heart transplantation [1]. However, their use is associated with significant long-term side effects. CNI nephrotoxicity is of particular concern. One in ten heart transplant recipients will ultimately develop end stage renal failure, which is associated with a more than fourfold increase in mortality [2, 3]. Furthermore, CNIs contribute to metabolic disturbances such as posttransplant diabetes mellitus, dyslipidemia, and hypertension and predispose to posttransplant malignancies and infection [4, 5]. Finally, CNIs do not prevent the development cardiac allograft vasculopathy (CAV) [6].

The availability of everolimus has sparked interest in the development of CNI-sparing and CNI-free immunosuppressive strategies. Everolimus is a derivative of sirolimus (rapamycin) and works similarly as a mammalian target of rapamycin (mTOR) inhibitor. It does not inhibit interleukin-2 production from antigen-induced T-cell activation but inhibits growth-factor induced cellular proliferation in response to alloantigens [7], hence the name “proliferation signal inhibitor.” Several characteristics make everolimus an attractive alternative for CNIs in heart transplant recipients. First, everolimus is not nephrotoxic. By reducing exposure to CNIs, it could potentially preserve renal function [1]. Second, everolimus restricts growth factor-dependent proliferation of vascular smooth muscle cells [8]. It has been shown to significantly decrease the incidence of CAV in de novo trials [9–12]. Third, everolimus interferes with the phosphatidylinositol 3-kinase pathway, a critical step for viral signaling and replication. There is convincing evidence indicating a reduced rate of cytomegalovirus (CMV) infection in everolimus-treated heart transplant recipients [13–15]. Fourth, everolimus exhibits antiproliferative activity. This has led to its licensing in the treatment of renal cell carcinoma and other tumors [16]. Although doses prescribed for malignancies are much higher, preliminary data suggest that everolimus-based immune suppression could decrease the incidence of certain post-transplant malignancies [17, 18].

However, the widespread implementation of everolimus in heart transplantation has been limited by several concerns. First, when combined with CNIs, everolimus seems to potentiate CNI nephrotoxicity, unless CNI dose is substantially decreased [1, 7, 19, 20]. Second, de novo CNI-free immunosuppression or early CNI withdrawal is associated with a higher rejection rate [21, 22]. Third, everolimus is poorly tolerated, especially at higher doses [19]. It is associated with a delayed wound healing, oral aphthosis, edema, pulmonary toxicity, bacterial infections, thrombocytopenia, hyperlipidemia, and proteinuria [23].

In a recent randomized trial [21] involving 115 de novo heart transplant recipients, an everolimus-based regimen with early calcineurin withdrawal was associated with a significant improvement in kidney function in comparison with conventional CNI-based therapy. Whether everolimus initiation and complete CNI withdrawal results in a better renal outcome in maintenance cardiac transplant patients with established renal dysfunction has not been investigated in randomized trials. The present CECARI study (Certican Initiation and Calcineurin Inhibitor Withdrawal in Maintenance Heart Transplant Recipients with Renal Insufficiency) was designed to assess this issue.

## 2. Materials and Methods

### 2.1. Study Design

**Figure 1:**
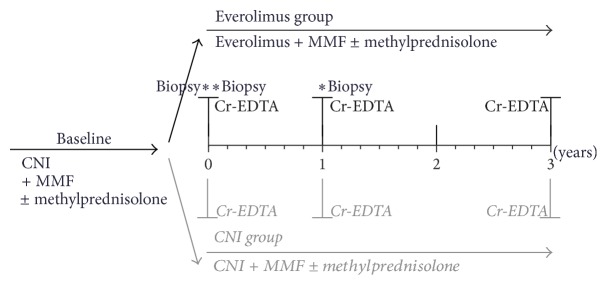
Study design. ^*∗*^protocol myocardial biopsy; CNI, calcineurin inhibitor; MMF, mycophenolate mofetil; Cr-EDTA, mGFR measurement by Cr-EDTA clearance.

CECARI was a three-year, prospective, multicenter, randomized, open-label trial, comparing everolimus initiation and CNI withdrawal, with conventional CNI-based therapy, in maintenance heart transplant recipients with renal dysfunction. The study was conducted between October 2007 and November 2013 at four transplant centers in Belgium. The study protocol was approved by the Institutional Review Board of each institution and conducted in accordance with Good Clinical Practice and the Declaration of Helsinki. All study participants provided written informed consent. The study was registered at clinicaltrialsregister.eu (reference number 2007-002102-22). The study design is shown in Figure 1.

### 2.2. Patients

To be eligible, patients had to be ≥18 years old, have undergone heart transplantation ≥1 year previously, receive standard CNI-based immunosuppression, and have moderate renal dysfunction (estimated GFR 30–60 mL/min/1.73 m^2^). Patients with an identifiable cause of chronic kidney disease other than CNI toxicity, treated acute rejection in the previous 6 months, malignancy within the last 5 years, HIV, hepatitis B or C infection, current severe systemic infection, current or planned pregnancy, severe thrombocytopenia (<75,000/*μ*L), leukopenia (<2500/*μ*L), anemia (Hb < 8 g/dL), hypercholesterolemia (≥350 mg/dL), hypertriglyceridemia (≥750 mg/dL), or proteinuria (≥0.8 g/24 h) were excluded.

### 2.3. Intervention

Patients were randomized in a 1 : 1 ratio between (i) switch to everolimus plus mycophenolate mofetil (MMF) with complete CNI withdrawal and (ii) continuation of their previous treatment with CNI plus MMF. Steroid use was left to the discretion of the treating physician, in both groups. In the everolimus group, CNI dose was reduced by 50% and everolimus was initiated at 0.75 mg twice daily. After obtaining an everolimus trough level between 6 and 8 ng/mL, CNI was discontinued. MMF was continued unchanged. In the CNI group, baseline treatment with CNI (either cyclosporine or tacrolimus) plus MMF was continued. Target trough levels and dose adjustments were left to the discretion of the treating physician.

### 2.4. Primary Endpoint, Efficacy, and Safety Assessment

The primary endpoint was change in measured GFR (mGFR) from baseline to year 3. The composite safety endpoint was all-cause mortality, treated acute rejection episodes, or major adverse cardiovascular events (MACE) at year 3. Secondary endpoints were change in mGFR at year 1, the individual subcomponents of the composite safety endpoint, proteinuria and lipid profile, tolerability and occurrence of infection, and other adverse events. mGFR was assessed by Cr-EDTA clearance at baseline, year 1, and year 3. MACE was defined by cardiac death, acute myocardial infarction, need for coronary revascularization, stroke, or admission for congestive heart failure. Protocol myocardial biopsies were performed before, one month and 12 months after CNI withdrawal in the everolimus group, and additionally when clinically indicated. Samples were evaluated locally according to the International Society of Heart and Lung Transplantation (ISHLT) criteria [33]. In the CNI group, myocardial biopsies were only performed on indication.

### 2.5. Statistical Analysis

The primary endpoint, change in mGFR from baseline to year 3, was compared between treatment groups using an analysis of covariance (ANCOVA) with the randomized group as a factor and the baseline value as a covariate. Continuous variables were presented as mean ± standard deviation or median and interquartile range (IQR), as appropriate. Categorical variables were presented using observed frequencies and percentages. Differences across groups were assessed using the Wilcoxon rank-sum test for continuous variables or the chi-square or Fisher's exact test for categorical variables, as appropriate. Time to event data were assessed by Kaplan-Meier statistics and compared using the log-rank test. For treated acute rejection and MACE, cumulative incidence functions were used to estimate event rates, whereby overall mortality was considered to be a competing risk. Groups were compared using the Pepe-Mori test. Efficacy analyses were conducted on all randomized patients who had data available. A post hoc on-treatment analysis was also conducted. A sample size of 50 patients (25 per treatment arm) was estimated to have 80% power to detect a mean ± SD difference between treatment groups of 8 ± 10 mL/min. Statistical significance for all analyses was set at a 2-tailed probability level of 0.05. Statistics were performed with the use of SAS software version 9.2 (SAS Institute Inc., Cary, NC) for Windows.

## 3. Results

### 3.1. Patients

**Figure 2:**
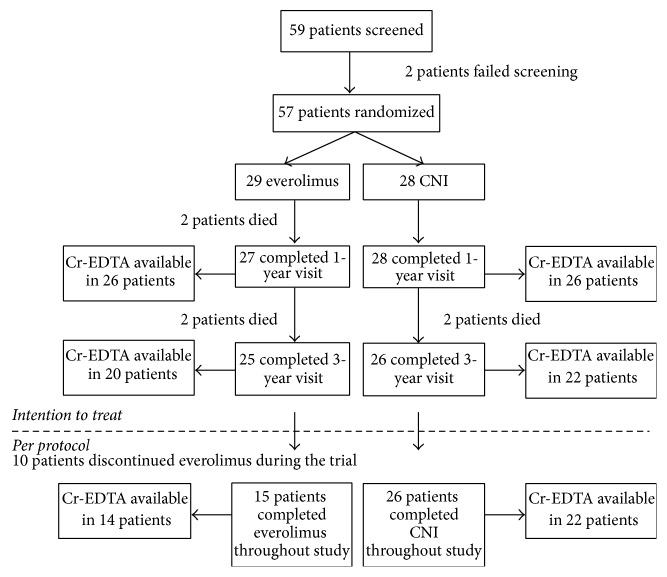
Study flowchart. CNI, calcineurin inhibitor; Cr-EDTA, measured glomerular filtration rate by Cr-EDTA clearance.

Fifty-nine patients were screened for participation. Two of them failed screening; thus, 57 patients were randomized: 29 to the everolimus group and 28 to the CNI group (Figure 2). Fifty-five completed the 1-year visit, and 51 completed the final 3-year visit; 6 patients died. No patients were lost to follow-up, but Cr-EDTA clearance at year 3 could not be obtained in 9 patients. Ten patients in the everolimus group discontinued study drug and were switched back to CNI (all during the first year); they were excluded in the post hoc on-treatment analysis. Mean age at randomization was 59.7 years (SD = 13.3). Time after heart transplantation was 1 to 18 years (median 7.0, IQR 4.0–12.6). Demographics and baseline characteristics did not differ significantly between both groups (Table 1). Except for a higher proportion of patients receiving loop diuretics in the everolimus group (44.8% versus 10.7%, *p* = 0.004), concomitant medication was similar. All patients received statin therapy.

### 3.2. Immunosuppression

At baseline, patients in both groups were treated with MMF and CNI, either cyclosporine (65.5% in everolimus group, 71.4% in CNI group, *p* = 0.63) or tacrolimus. Forty-nine percent of patients were treated with low dose methylprednisolone (51.7% in everolimus group, 46.4% in CNI group, *p* = 0.69). Throughout the study, mean everolimus trough levels were between 5.2 and 6.7 ng/mL, which is a slightly lower than the intended 6–8 ng/mL. In the CNI group, average cyclosporine and tacrolimus trough levels were between 103 and 122 ng/mL and between 7.5 and 9.1 ng/mL, respectively.

### 3.3. Renal Function

**Figure 3:**
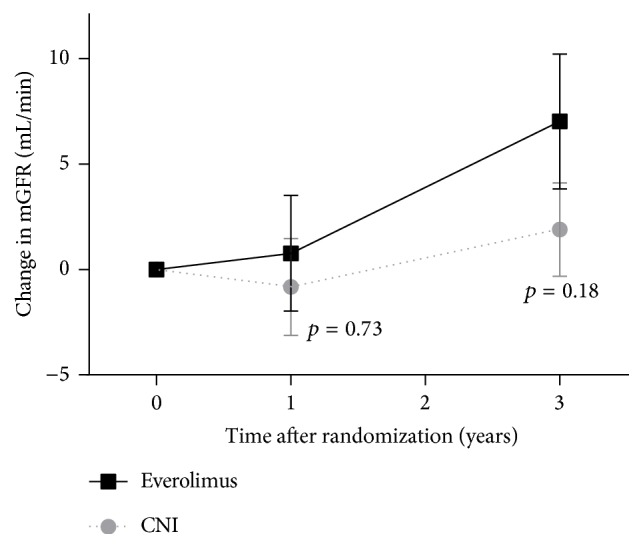
Mean (SEM) change in measured GFR from baseline. CNI, calcineurin inhibitor; SEM, standard error of the mean.

At baseline, mean mGFR was similar in both groups (38.5 mL/min (SD = 12.8) in the everolimus group and 39.3 mL/min (SD = 11.2) in the CNI group, *p* = 0.38). The change in mGFR did not differ significantly between both groups after 1 year (+0.76 mL/min (SD = 13.8) in the everolimus group, −0.83 mL/min (SD = 11.68) in the CNI group, *p* = 0.73). After 3 years, mGFR increased by a mean of 7.0 mL/min (SD = 14.9) in the everolimus group and by 1.9 mL/min (SD = 10.4) in the CNI group, but the difference was not significant either (*p* = 0.18) (Figure 3). A post hoc analysis was performed to evaluate the change in renal function after exclusion of the patients that discontinued everolimus. In this on-treatment analysis, the change in mGFR from baseline to year 3 did reach statistical significance (+9.4 mL/min (SD = 16.1) in the everolimus group versus +1.9 mL/min (SD = 10.4) in the CNI group, *p* = 0.047).

### 3.4. Immunosuppressive Efficacy

There was no difference in the composite endpoint of death, treated acute rejection or MACE (31.0% versus 25.0%, *p* = 0.50, Table 2). Individual components of the composite safety endpoint were also similar, except for a trend towards more treated rejection episodes in the everolimus group (10.3% versus 3.6%, *p* = 0.09). These rejections occurred early after switch from CNI to everolimus: after 33 days, 68 days, and 371 days. None of these rejections resulted in graft loss. There were 4 deaths in the everolimus group (lung cancer, sudden cardiac death, heart failure, and septic shock) and two in the CNI group (lung cancer and sudden cardiac death) (*p* = 0.38).

### 3.5. Safety and Tolerability

There were significantly more adverse events in the everolimus group (96.6 versus 57.1%, *p* < 0.001, Table 3). The most common adverse events were infection, anemia, lower extremity edema, and skin rash. There was no significant difference in proteinuria (0.32 g/L in the everolimus group versus 0.16 g/L in the CNI group, *p* = 0.40) or total cholesterol (168.7 versus 174.0 mg/dL, *p* = 0.70). Malignancy occurred in 6.9% in the everolimus group versus 10.7% in the CNI group (*p* = 0.67). Study drug was discontinued in 10 patients (34.5%) of the everolimus group due to adverse events. There were no study drug discontinuations in the CNI group.

## 4. Discussion

The management of posttransplant renal dysfunction is challenging. The widely recognized nephrotoxicity associated with CNIs has prompted the search for everolimus-based CNI-sparing and CNI-free regimens. However, after more than a decade of experience with everolimus in heart transplantation, the optimal strategy is still unknown.

The present CECARI study was the first to assess whether an everolimus-based CNI-free strategy improves renal function in maintenance heart transplant recipients with established renal insufficiency, compared with conventional CNI-based therapy. The key findings of this small, prospective, randomized, multicenter study were as follows: (i) everolimus initiation and CNI withdrawal in maintenance heart transplant recipients did not lead to a significantly better renal outcome; (ii) while feasible without loss of efficacy, adherence to the everolimus regimen was relatively poor due to adverse events; (iii) the selected patients that tolerated everolimus (on-treatment analysis) did have a better renal outcome.

Everolimus has been investigated in de novo and maintenance heart transplant recipients before, as both part of a CNI-sparing (dose reduction) and a CNI-free (complete CNI withdrawal) strategy, with varying results on kidney function (Table 4). Eisen et al. [19] compared everolimus with azathioprine, both in combination with standard exposure cyclosporine, in de novo heart transplantation. While showing superior efficacy and benefit on CAV development, everolimus was associated with a worse renal function; this was attributed to the potentiation of cyclosporine nephrotoxicity by everolimus. Lehmkuhl et al. [24] and Eisen et al. [20] compared everolimus plus reduced exposure cyclosporine, with standard exposure cyclosporine plus MMF. None of these trials could show a renal benefit of the everolimus-based CNI-sparing strategy. Zuckermann et al. [25] and Wang et al. [26] investigated everolimus plus reduced exposure cyclosporine versus everolimus plus standard exposure cyclosporine but could not show a significant difference in renal function, either. In contrast, a CNI-free regimen of everolimus plus MMF did improve renal function in comparison with cyclosporine plus MMF in a trial of 115 de novo heart transplant recipients in SCHEDULE [21].

Three randomized trials have investigated everolimus-based CNI-sparing strategies in maintenance heart transplant recipients. The SHIRAKISS [31] trial and the study of Bara et al. [32] compared everolimus with MMF, both in combination with reduced exposure cyclosporine, but could not show a better renal outcome of the everolimus strategy; there even was a trend towards benefit of the MMF strategy. In contrast, NOCTET [29], a randomized trial of 282 maintenance thoracic transplant recipients (190 heart, 92 lung transplants), comparing everolimus plus reduced exposure CNI plus MMF with standard exposure CNI plus MMF, showed significant improvement in renal function at one year in the everolimus-group. The present study was the first randomized trial investigating an everolimus-based CNI-free (instead of CNI-sparing) regimen in maintenance heart transplant recipients. Sirolimus, another mTOR inhibitor, has been investigated in a CNI-free regimen in maintenance heart transplant recipients by Zuckermann et al. [34], showing improved renal function compared with the CNI-group, but, analogous to our study, a high discontinuation rate attributable to adverse events.

There are several possible explanations for the lack of a significant renal benefit in the everolimus group in our study. First, the median time after transplantation was long: 7.0 years. CNI elimination appears to be most effective in the first years after heart transplantation [35, 36]; patients with longstanding CNI exposure are less likely to benefit. In NOCTET, the benefit of CNI reduction in maintenance thoracic transplant patients was limited to those less than five years after transplantation [37]. This probably reflects established and irreversible renal damage. Second, the everolimus discontinuation rate was high; this might have masked a potential benefit of CNI withdrawal on renal function. The on-treatment analysis suggests that patients who adhere to the everolimus-based regimen do have a better renal outcome. Third, although the two groups were relatively well matched, there was a substantial difference in loop diuretic use: 44.8% in the everolimus group versus 10.7% in the CNI group, *p* = 0.004. This could have influenced renal function in the everolimus group in a negative way.

The current results indicate that everolimus initiation and complete CNI withdrawal in maintenance heart transplant recipients is feasible. The composite endpoint of all-course mortality, MACE, and treated acute rejection was similar in both groups. There was a trend towards a higher rate of treated acute rejection, but this did not lead to graft loss. However, patients in the everolimus group had significantly more adverse events. These mostly occurred during the first three months and were most often benign and nonfatal (lower extremity edema, oral aphthosis) but unfortunately led to a high everolimus discontinuation rate.

The current study does not support a universal use of everolimus for kidney protection in maintenance heart transplant recipients. Rather, it suggests benefit in selected patients. Other studies have found that patients at less than five years after transplant [37] and those without baseline proteinuria [31] are most likely to benefit. Future studies should focus on these patients. Above all, they should try to improve everolimus adherence. Most adverse events in our trial occurred early after switching to everolimus. A lower starting dose, followed by gradual everolimus up-titration and concomitant CNI down-titration, might therefore improve tolerance. Finally, growing clinician's experience using everolimus and managing its adverse events could potentially further reduce the discontinuation rate.

The present study had several limitations. First, the number of study drug discontinuations was high. This may have influenced the primary endpoint, as the on-treatment analysis did show a significantly greater improvement in renal function. Second, mean everolimus trough level was slightly lower than the predefined target. However, this was not associated with a loss of immunosuppressive efficacy. Third, protocol myocardial biopsies were performed at baseline, one month, and one year in the everolimus group, whereas biopsies were only performed on indication in the CNI group. A detection bias for treated acute rejection episodes can therefore not be excluded.

In conclusion, the present study did not show a significantly better renal outcome of everolimus initiation and CNI withdrawal in maintenance heart transplant recipients. However, poor adherence to the everolimus regimen meant that the potential benefit of CNI withdrawal could not be fully evaluated. Future protocols should consider measures to improve everolimus adherence.

## Figures and Tables

**Table 1:** Baseline characteristics.

	Everolimus (*N* = 29)	CNI (*N* = 28)	*p* value
Age (years), mean ± SD	61.0 ± 14.9	58.3 ± 11.5	0.46
Female, *n* (%)	2 (6.9)	1 (3.6)	1.0
Ethnic origin, Caucasian, *n* (%)	29 (100)	28 (100)	1.0
Time posttransplant (years), median; IQR	7.6; 4.1–12.9	6.5; 3.7–12.1	0.82
Medical history, *n* (%)			
Hypertension	24 (82.8)	27 (96.4)	0.19
Diabetes mellitus	7 (24.1)	3 (10.7)	0.30
Laboratory values, mean ± SD			
mGFR (mL/m)	38.5 ± 12.8	39.3 ± 11.2	0.38
Creatinine (mg/dL)	1.74 ± 0.28	1.66 ± 0.29	0.19
eGFR (mL/min/1.73 m^2^)	41.6 ± 8.1	45.1 ± 7.6	0.09
Urea (mg/dL)	83.7 ± 28.2	74.1 ± 19.4	0.28
Total cholesterol (mg/dL)	161.2 ± 30.1	165.3 ± 43.0	0.95
Proteinuria (g/L)	0.12 ± 0.09	0.09 ± 0.07	0.20
Immunosuppression, *n* (%)			
Cyclosporine	19 (65.5)	20 (71.4)	0.63
Tacrolimus	10 (34.5)	8 (28.6)	0.63
MMF	29 (100)	28 (100)	1.0
Methylprednisolone	15 (51.7)	13 (46.4)	0.69
Concomitant medication, *n* (%)			
ACE-inhibitors	13 (44.8)	18 (64.3)	0.14
ARB	4 (13.8)	4 (14.3)	1.0
Loop diuretics	13 (44.8)	3 (10.7)	0.004
Spironolactone	3 (10.3)	0	0.24
Statins	29 (100)	28 (100)	1.0

SD, standard deviation; MMF, mycophenolate mofetil; ARB, angiotensin receptor blocker.

**Table 2:** Safety endpoints at year 3.

	Everolimus (*N* = 29)	CNI (*N* = 28)	*p* value
Composite endpoint, *n* (%)	9 (31.0)	7 (25.0)	0.50
Death	4 (13.8)	2 (7.1)	0.38
Treated acute rejection	3 (10.3)	1 (3.6)	0.09
MACE	5 (17.2)	6 (21.4)	0.96

MACE, major adverse cardiovascular event; CNI, calcineurin inhibitor.

**Table 3:** Adverse events at year 3.

	Everolimus (*N* = 29)	CNI (*N* = 28)	*p* value
Any adverse event, *n* (%)	28 (96.6)	16 (57.1)	<0.001
Study drug discontinuation	10 (34.5)	0	<0.001
Infection	19 (65.5)	14 (50.0)	0.24
Infection with need for hospitalization	10 (34.5)	4 (14.3)	0.077
Neoplasm	2 (6.9)	3 (10.7)	0.67
Anemia	10 (34.5)	0	0.001
Leukopenia	6 (20.7)	1 (3.6)	0.10
Thrombocytopenia	1 (3.4)	0	1.0
Lower extremity edema	10 (34.5)	2 (7.1)	0.011
Skin rash	8 (27.6)	0	0.004
Oral aphtosis	5 (17.2)	0	0.052
Pulmonary toxicity	5 (17.2)	0	0.052
Diarrhea	7 (24.1)	2 (7.1)	0.14

CNI, calcineurin inhibitor.

**Table 4:** Comparison of randomized trials of everolimus in heart transplant recipients.

	*N*	FU	Timing^*∗*^	Intervention	Control	Renal function	
						Baseline	Effect of intervention
(a) de novo							
*CNI-sparing*							
Eisen et al. [19]	634	1 y	at Tx	SE CsA + EVL	SE CsA + AZA	NA	EVL *worse*
Lehmkuhl et al. [24]	176	1 y	at Tx	re CsA + EVL	SE CsA + MMF	eGFR 74.7	No significant difference
Eisen et al. [20]	721	1 y	at Tx	re CsA + EVL	SE CsA + MMF	eGFR 66.8	EVL *worse*
Zuckermann et al. [25]	199	6 m	at Tx	re CsA+ EVL	SE CsA + EVL	SCr 1.3	No significant difference
Wang et al. [26]	25	6 m	at Tx	re CsA + EVL	SE CSA + EVL	SCr 1.1	No significant difference
*CNI-free*							
SCHEDULE [21, 27]	115	3 y	+7 w	EVL + MMF	SE CsA + MMF	SCr 1.2	EVL *better*
MANDELA [28]	200	1 y	+6 m	EVL + MMF	re CNI + EVL	NA	Currently ongoing

(b) Maintenance							
*CNI-sparing*							
NOCTET [29, 30]	190^†^	5 y	+6.3 y	re CNI + EVL + MMF/AZA	SE CNI + MMF/AZA	mGFR 47.6	EVL *better*
SHIRAKISS [31]	34	3 y	+2.6 y	re CsA + EVL	re CsA + MMF	CrCl 43.9	No significant difference
Bara et al. [32]	70	1 y	+4.8 y	re CxA + EVL	re CsA + MMF	SCr 2.1	No significant difference
*CNI-free*							
CECARI (present study)	57	3 y	+7.0 y	EVL + MMF	re CNI + MMF	mGFR 38.9	No significant difference

AZA, azathioprine; CNI, calcineurin inhibitor (cyclosporine or tacrolimus); CsA, cyclosporine A; EVL, everolimus; MMF, mycophenolate mofetil; re, reduced exposure; SE, standard exposure; Tx, transplantation.

CrCl, creatinine clearance (mL/min); eGFR, estimated GFR (mL/min/1.73 m^2^); mGFR, measured GFR (mL/min); NA, not available; SCr, serum creatinine (mg/dL).

^*∗*^Timing of intervention relative to transplantation; FU, longest available follow-up; w, week; m, month; y, year.

^†^In NOCTET, a total of 282 patients were included: 190 heart transplant + 92 lung transplant recipients.
